# Contemporary Endodontic Approaches for Children

**DOI:** 10.3390/clinpract13040083

**Published:** 2023-08-03

**Authors:** Alfredo Iandolo

**Affiliations:** Department of Medicine, Surgery and Dentistry, University of Salerno, 84084 Salerno, Italy; aiandolo@unisa.it

Endodontic therapy is necessary when caries extend further into the tissues of the tooth and reach the pulp, producing irreparable inflammation or necrosis [1,2]. Numerous conditions, like dental trauma, persistent irritation, and deep cavities, can contribute to pulp inflammation [3].

Due to patients’ desire to keep their original teeth and a growing understanding of the advantages of retaining natural teeth, this dental operation is becoming increasingly common.

The success of an endodontic treatment depends on eliminating damaged or necrotic tissues, bacteria, and accumulated hard tissue debris [4,5]. Regardless of the instrumentation method, over 30% of the root canal area is untrimmed. It is crucial to undertake chemical preparation by activating the irrigants [6,7,8].

Success in endodontics is based on proper shaping [9,10], three-dimensional cleaning [11,12], and the 3D obturation of the complex root canal space [13,14,15]. The endodontic management of teeth with immature apexes is a problem due to the various difficulties that may occur during the treatment [16,17]. A tooth with an immature apex may be treated using several techniques, including indirect pulp capping, direct pulp capping, partial pulpotomy, full pulpotomy, apexification, and an apical plug [18,19,20]. The current research focuses on regenerative endodontic procedures, which are biological techniques used to repair damaged dentin, root structures, and cells with complex pulp–dentinal structures. Apexification might be replaced with a new therapeutic strategy called revascularization [21,22,23].

Additionally, the revascularization of developing permanent teeth with necrotic pulp infection and apical periodontitis or abscesses is encouraged. To that aim, radiographic findings revealed a thickening of the root canal walls and ongoing root development in immature permanent teeth with apical periodontitis undergoing revascularization [24]. Compared to other procedures, the regenerative approach has some important advantages: complete root development leads to a greater resistance of the element to fracture and a longer survival time in the oral cavity [25,26]. Of course, the flawless execution of every protocol is required for success. Everything must be carried out in a sterile setting, from the isolation of the operating field to active cleaning [27,28,29,30,31].

In conclusion, the proper implementation of current clinical protocols, materials, and technologies will assure the success of endodontic treatments such as regenerative approaches.
